# Adult women identities on the menu: deconstructing fast-food consumption among university students

**DOI:** 10.1186/s40795-026-01287-3

**Published:** 2026-05-05

**Authors:** Basma W. Elrefay, Shadia M. Bassiouny, Omnia Ragab Mohamed Hammouda, Doaa Hamed Elsabakhawi, Ghalia E. Elkasaby, Amina Mohamed Rashad El-Nemer

**Affiliations:** 1 Lecturer of Obstetrics and Gynecology Health Nursing, Faculty of Nursing, Delta University for Science and Technology, Gamasa, 11152 Egypt; 2 Alghad College for Applied Medical Sciences, Jeddah, Saudi Arabia; 3 Batterjee Medical College, Jeddah, Saudi Arabia; 4 National Nutritional Institute, Cairo, Egypt; 5 Urology & Nephrology Centre, Mansoura University, Mansoura, Egypt; 6 Faculty of Nursing, Delta University for Science and Technology, Gamasa, Egypt

**Keywords:** Health beliefs, Dietary habits, Lifestyle factors, Social influence, Nutrition knowledge, Weight management

## Abstract

**Background:**

Fast food is a highly prevalent choice of diet for Female university students because of its convenience and Affordability. Whichever the reason, good nutrition is vital for the best health and well-being. The issue is that, although the adequate intake of nutrients is crucial to health, relying too much on fast foods can lead to poor health consequences. The eating behavior of university women is usually influenced by social influences, time constraints, and financial limitations. This study investigated factors correlating with fast-food consumption and their relation to identity formation among 385 female university students in Egypt.

**Methods:**

A mixed-methods approach was used involving both survey and focus group techniques. These methods involved A survey that included participants’ sociodemographic, eating habits, fast food knowledge, and attitudes about consumption; control perception related behaviors; subjective norms; and intentions.

**Results:**

showed moderate awareness regarding risk factors associated with fast food (mean = 5.37 ± 1.74), though 67.8% considered fast food to be tasty. That awareness of health risks does not necessarily diminish its appeal. Attitudes toward fast food are moderately favorable (mean = 92.63 ± 15.06), though 57% believe fast food causes obesity. There was a significant relationship between perceived behavioral control (mean = 58.39 ± 9.47), which was influenced by craving and lack of time (*p* < 0.01). The subjective norms indicated that 74.3% considered family members’ approval important (*p* < 0.05). The result showed that intentions to reduce fast food behavior are moderately favorable (mean = 30.22 ± 3.52). The result showed that obese subjects scored high values for all constructs compared to nonobese subjects (*p* < 0.01). Family income was found to positively correlate with values for all constructs (*p* < 0.05), though there was no significant association between parental education (*p* > 0.05). High values positively associated with attitude (*r* = 0.826), attitude (*r* = 0.331), perceived behavioral control (*r* = 0.202), and subjective norms (*r* = 0.253), which indicated association between attitude and intention (*p* < 0.001), attitude and intention (*p* < 0.01), perceived behavioral control (*p* < 0.05), subjective norms (*p* < 0.01), respectively.

**Conclusion:**

From these results, it appears that interventions based on attitude, perceived behavioral control, or social influences—focusing on self-efficacy-enhancing or socially influenced eating behavior—may prove to be more effective in encouraging healthier eating behaviors in female university-age students.

**Trial registration:**

Our study was registered retrospectively with Clinicaltrials.gov under the identifier NCT06783959 on 29 January 2025.

**Supplementary Information:**

The online version contains supplementary material available at 10.1186/s40795-026-01287-3.

## Introduction

Fast foods are considered to be a hallmark feature of global diets because fast foods are rapidly increasing in popularity due to numerous benefits associated with them. They are not only considered to be tasty but also affordable. They are a preferred source of nutrition for young people who are in college because they are convenient to consume [1]. Observational studies conducted in Egypt showed that consuming fast food was highly prevalent in colleges. It was specifically found that female college goers prefer to eat fast food. Several observations made in Alexandria University reported that eating fast food was a common feature amongst female college goers. These observations emphasize that fast foods are gradually increasing in popularity amongst local populations [2]. The increasing use of fast foods amongst college goers has serious repercussions because these foods are rich in calories but lack nutrients. These eating habits are common amongst women who are in college. They are having difficulty juggling their personal, academic, and developmental lives [3]. These women are expected to face serious health problems because consuming fast-food leads to adverse reactions in them due to excess calorie intake [4].

Healthy eating has become more mainstream, but so has fast food. Healthy meals that are rich in nutrients like fruits, vegetables, grains, and proteins are considered essential for healthy living because these meals are rich in crucial nutrients [5]. Consuming healthy meals every day can improve vitality, improve cognitive functions, and reduce risks associated with chronic diseases [6]. But women find it extremely challenging to adapt to a healthy diet because women are not only deeply influenced socially because of eating behaviors and preferences, but are also struggling to manage eating according to “their idea of self.” Women’s eating behaviors are directly related to self-construction because eating fast foods in particular offers them “an opportunity for self-expression” related to eating behaviors in particular [7]. Women use fast food to construct “their sense of self.” Therefore, understanding these eating behaviors related to adult and adolescent eating preferences is crucial because these behaviors related to eating fast foods are not merely related to health but are directly associated with women’s construction related to eating behaviors [8]. female university students continue to consume fast foods because of reasons like “socialization, trends, time constraint, and economic conditions [9]. In universities or colleges in particular, fast foods become “the most convenient and cheapest alternative.” These behaviors related to fast foods continue to promote eating fast foods because “peer eating behaviors and need for social affiliation are influential., Women’s eating behaviors related to fast foods are not merely related to health because these eating behaviors are directly related to women’s construction because women continue fast food consumption to promote “their self-expression [10]. furthermore, regular consumption can be directly related to women’s construction because considering “societally defined value for appearance,” eating behaviors related to fast foods directly influence women’s construction because eating fast foods continue to “negatively affect women’s mental well-being [11].

With these complex forces in mind, describing the behavioral and cognitive antecedents that regulate fast food intake in young women assumes great significance. In terms of public health nursing, health education regarding nutrition issues can prove to be a vital method to prevent related problems. In light of these observations, health educators like nurses can prove to be essential allies who can sensitize young women to exercise healthy nutritional behavior with a deeper understanding of psychosocial forces that define these behaviors. Nurses can help to define future courses to promote nutritional well-being in women [12].

Although there are numerous studies related to fast food consumption, there are fewer studies related to the psychosocial-behavioral predictors of fast-food choices made specifically by female university students in Egypt. The current study seeks to address that gap. Understanding these factors through the Theory of Planned Behavior can provide useful information for future nursing interventions [13, 14].

### Aim of the study

This study aimed to explore the factors related to fast food consumption in female university students and its association with the formation of identity. In particular, this study attempted to explore the role of knowledge, attitude, perceived control belief, and subjective norms in fast food consumption behavior and the formation of identity in female university students. This has particular significance to nursing interventions related to healthy eating behaviors.

### Significance of the study

This study addresses an underexplored area of fast-food consumption and identity formation among female university students in Egypt. The inclusion of participants from different faculties ensured diverse perspectives, while the use of validated tools enhanced the reliability of the findings. Beyond confirming existing evidence on convenience and affordability, the study identified distinct psychosocial factors such as self-concept, peer influence, and social affiliation as major predictors of fast-food consumption among female university students. These insights extend previous research and offer new implications for nursing-led health education and behavioral interventions. However, the study relied on self-reported data, which may have introduced recall or social desirability bias. Its cross-sectional design also limits causal inference. Furthermore, psychosocial factors such as stress or self-image were not explored in depth.

### Study hypothesis

Primary Hypothesis: Fast food eating behavior within female university students has a positive association with the expression of identity, and their Food choices are reflective of these females’ self-perceptions.

Secondary Hypothesis: Social dynamics like peer pressure or culture-related stories greatly influence fast food eating behaviors among female university students.

## Materials and methods

### Study design

A mixed-methods design was used in this study; both quantitative and qualitative methods were utilized to address fast food consumption behaviors in relation to the formation of female university student identities. Quantitative data were collected using a structured survey to measure data based on fast food consumption behaviors, knowledge, attitudes, perceptions of behavior control, subjective norms, and intentions. Qualitative data were obtained using focus group procedures to measure subjective perspectives related to formation or attitudes related to fast food consumption behavior.

The ethical requisites of the Helsinki Declaration (1975) were considered in carrying out this research. Also, this study was conducted between 21 May 2024 to 12 January 2025. In fact, prior approval to conduct the study was given in written format by the Research Ethics Committee in the Faculty of Nursing at Mansoura University (IBR: 0692 dated 4 Jan 2025). Although retrospective data collection was involved in carrying out the study, the study was merely registered with ClinicalTrials.gov (NCT06783959) dated 29 Jan 2025.

### Study settings

The study was conducted at Mansoura University in Egypt. The target participants were female students in grades 1 to 4 in four faculties: two practical faculties (the Faculty of Science and Agriculture), and two theoretical faculties (the Faculty of Education and Arts). These faculties were specifically chosen to get a well-variety sample. Both scientific and theoretical subjects studied in faculties can possibly affect eating behavior, attitudes, and identity-related elements. Representing a range of academic levels and age groups.

### Participants

The study targeted 385 female students aged between 18 and 24 years in the targeted colleges. These are students in grades 1–4 who agreed to participate in the study.

Study settings.

### Sample size calculation

Sample size calculation was performed with the G*Power software based on the formula for estimating proportions:

$$\mathrm{n}=(\mathrm{Z}^\wedge2{^*}\mathrm{p}^*(1\mathrm{p}))/\mathrm{e}^\wedge2$$

$$\mathrm{n}=(\mathrm{Z}^2{^*}\mathrm{p}^*(1-\mathrm{p}))/\mathrm{e}^{2}$$

where:

n = sample size

Z = Z-score corresponding to the desired confidence level (1.96 for 95%)

p = estimated population proportion

e = desired margin of error

The calculated sample size was about 385 participants. To ensure statistical power, 10% more were targeted (*n* = 425) for participation, taking into account possible non-response cases. After data cleaning, a total of 400 fully completed questionnaires were included in the final data analysis, and 25 incomplete responses were excluded.

### Inclusion criteria

This study included female students who were enrolled in one of four selected faculties from Mansoura University, within the age limit of 18 to 24 years. These faculties are: the Faculty of Science, the Faculty of Agriculture, the Faculty of Education, and the Faculty of Arts. Participants in the research were randomly selected from all academic grades, which ranged from grade 1 to grade 4, so that each educational level and age group was equally represented. Informed consent to participate in the present research was obtained on a volunteer basis from all participants, who agreed and declared their willingness to fill in the survey questionnaire and participate in the research.

### Exclusion criteria

These studies excluded any male students, and female students from those younger than 18 or older than 24 years, and who were not enrolled in one of the selected faculties in Mansoura University were also excluded from participation. Other exclusion criteria included students with medical disorders, like eating disorders and metabolic disorders, or students following a specific medically prescribed diet that might affect the results. Lastly, exclusion was applied to anyone who had participated in any research related to fast food consumption and identity building, to keep the sample fresh and reduce biases in this study.

### Study tools

- *Part I: Socio-demographic data* including age, body mass index (BMI), parents’ education and employment status, family income, health interest, and body weight management. BMI was computed from the self-reported height and weight. The questionnaire was developed to answer the study aims, and after a review of the relevant literature, and was not published elsewhere before. The English versions of these tools were provided as a supplementary file.
- *Part II: Qualitative Semi-structured interview Guide of eating habits)* The guide consisted of open-ended questions that examined participants’ dining practices and habits of consuming fast food. The questions probed into aspects such as frequency, time use, expenditure, social setting, and preference. Respondents were encouraged to expand on their experiences and motivations such that an in-depth qualitative insight into the fast-food dining habit was possible. An interview guide questionnaire was developed based on the study objectives and review of the relevant literature, and wasn’t published elsewhere. The English versions of these tools are provided as a supplementary file.
- *Part III: Knowledge of fast-food consumption*: This part, adapted from Didarloo et al. (2022) [15], consists of 14 items assessing knowledge about fast food, its side effects, and principles of healthy nutrition.

Scoring system

Knowledge of fast-food consumption: Scoring of the knowledge part was then revised, whereby “No” or “I don’t know” responses were given a score of 0, and “Yes” responses were given a score of 1. Thus, the total possible score ranged from 0 to 14, where higher scores reflect a greater level of knowledge.

- *Part IV: Attitude towards fast food consumption*: This section measured participants' attitudes towards consuming fast food based on a multi-dimensional, comprehensive questionnaire. It consists of 28 items adapted from the validated tool by Didarloo et al. (2022) [15]. Items were included that would assess different components of attitudes related to the consumption of fast food: satisfaction, convenience, affordability, time efficiency, social influences, habituation, and concerns about environmental protection.

Scoring System

The items were rated on five-point Likert scales ranging from "Strongly Agree" through "Strongly Disagree", or "Very Important" through "Not At All Important", depending on the item. The individual item scores were from 1 through 5, with higher scores reflective of more favorable attitudes or higher importance attributed to that factor. The total score for this subscale could range from a minimum of 28 to a maximum of 140, with higher overall scores representing more favorable attitudes about their consumption of fast food.

- *Part V: Perceived Behavioral Control*: This section assessed participants made to their perceived behavioral control over fast-food consumption, using 18 items adapted from a validated instrument by Didarloo et al. (2022). Perceived behavioral control was measured with two separate subdimensions: control beliefs and perceived power. Control beliefs were assessed with nine items that asked participants about their beliefs regarding factors or conditions that work for or against one's ability to control fast food consumption. The items assessed personal, social, and environmental factors, including but not limited to the presence of fast foods, time pressure, and social pressure. Perceived power was assessed by the second set of nine items that asked participants the level they believed they would be able to overcome those barriers or if they would make choices that were in line with their intention.

Scoring system

Each item was scored on a five-point Likert-type scale ranging from “Strongly Agree” (5) to “Strongly Disagree” (1). Higher scores indicated greater perceived behavioral control over fast-food consumption. The total score in this section ranged from 18 to 90, with the total score indicating participants’ degree of control over fast-food consumption.

- *Part VI: Subjective norms*: The subjective norms section evaluated the participants' perception of social influences and expectations concerning their fast-food consumption. This component was made up of 10 items adapted from the tool by Didarloo et al. (2022) [15]. Items were aimed to assess two subdimensions: normative beliefs and motivation to comply.

Normative Beliefs: In this subdimension, five items were used to assess the participants' beliefs that important people or groups in their lives (e.g., friends, family members, or society as a whole) approve/disapprove of their eating fast food.

Motivation to Comply: This subdimension included five items that assessed the degree to which participants would be willing to conform to these perceived expectations or opinions in making decisions about fast food consumption.

In the current study, items 9 and 10, dealing with the wife's opinion and influence, were excluded because they were not appropriate for the study population. These two items were excluded to make the tool relevant, appropriate, and culturally acceptable for the target demographic.

Scoring system

The other eight were rated on a five-point Likert scale, with response options ranging from "Strongly Agree" to "Strongly Disagree" or from "Very Important" to "Not at All Important," depending on the context of the item. The full range of scores for subjective norms was between 8 and 40, where higher scores indicated the presence of strong perceived social influences and motivation to conform to such influences.

- *Part VII: Behavioral Intention Assessment Questionnaire:*The section on behavioral intention sought to evaluate participants’ intentions with regard to consuming fast food prime predictor of future behavior, according to TPB. According to TPB, human behavior is influenced by three major determinants: attitude towards the behavior, which is the individual’s positive or negative evaluation of the behavior; subjective norms, or the perceived social pressure to perform or not perform the behavior; and finally, perceived behavioral control, or the perceived ease or difficulty of performing the behavior. These factors jointly shape behavioral intention, which in turn predicts actual behavior (Ajzen, 1991) [16]. This segment of the instrument was an eight-item scale adapted from Didarloo et al. (2022) [15] for assessing how intense the intentions of the participants were to consume or avoid fast foods in the future. Each item in this segment was developed to assess the likelihood of engaging in fast food consumption based on factors as frequency, context, and convenience. Items measured self-reported plans and readiness of the participants to engage in fast food consumption and attitudes about the benefits or detriments of such behaviors.

Scoring system

The Behavioral Intention Assessment had a five-point Likert scale, whereby the answers ranged from “Strongly Agree” with 5 points to “Strongly Disagree” with 1 point. Hence, the minimum mark for the behavioral intention section was set at 8, while the maximum was at 40 points. Thus, the higher the score, the greater the intentions to consume fast food in the future.

### Pilot study

A pilot study was conducted with 10% of the final sample size to test the feasibility, reliability, and clarity of the study tools. Feedback was used to revise the tools for the main study.

### Reliability

Internal consistency was evaluated by using Cronbach’s alpha coefficient; its value was acceptable if it was 0.70 and above. The Cronbach’s alpha values of the subscales were as follows: Knowledge of Fast-Food Consumption (α = 0.75), Attitude towards Fast Food Consumption (α = 0.82), Perceived Behavioral Control (α = 0.88), Subjective Norms (α = 0.81), and Behavioral Intention (α = 0.79). The values reflected satisfactory to excellent internal consistency for all subscales. To establish test–retest reliability, the questionnaire was completed twice by a subsample of participants, separated by two weeks. The test-retest correlation coefficients were as follows: Knowledge of Fast-Food Consumption (*r* = 0.85), Attitude toward Fast Food Consumption (*r* = 0.90), Perceived Behavioral Control (*r* = 0.91), Subjective Norms (*r* = 0.82), and Behavioral Intention (*r* = 0.87), which indicates the instruments have very high stability and reliability over time.

### Validity

In validating the study instruments, content and construct validities were established. The content validity was established by a panel of five experts in nutrition, health behavior, and social sciences who examined the questionnaire to establish whether the items adequately represented the key concepts in this study. The experts provided feedback leading to revisions of the items sufficiently reflecting fast-food consumption, identity formation, and social influences. The construct validity was established by examining the relationships between the subscales and the theoretical constructs. The correlations between the subscales provide evidence that the subscales are measuring related, but distinct, features of fast-food consumption and associated influences.

### Adaptation process

The questionnaires were initially developed by Didarloo et al. in 2022 and have undergone adaptation for the Egyptian context using a systematic cross-cultural validation process [15]. Two bilingual nursing experts familiar with the study constructs translated the questionnaires from English to Arabic. An independent back-translation into English was done by a language professional who was blinded to the original versions to ensure conceptual equivalence. Two bilingual nursing experts, along with members of the expert panel (of nursing and nutrition, as mentioned above), reviewed and resolved any discrepancies to ensure linguistic accuracy and cultural relevance. A pilot test was conducted with 10% of the intended sample to assess clarity, comprehensibility, and contextual relevance. Minor adjustments to wording and examples were made according to participants’ feedback, to enhance cultural relevance and appropriateness, although the original constructs were not altered. The expert panel approved the final Arabic version before it was distributed for the main study.

### Study procedures

Careful preparation was taken to carry out these study procedures in an ethical, responsible, and systematic way. The study procedures were composed of three phases: the preparatory phase, the participant recruitment phase, and the data collection phase.

#### Preparatory phase

Ethical approval to conduct this study was first received from the Institutional Review Board (IRB), Mansoura University. The IRB ensured that all ethical principles to protect human subjects in research were addressed. Next, the study tools were pilot tested for clarity, feasibility, and understandability. Participants made up of 10% of the required sample size were multi-stage sampled randomly from the target population of completion of the questionnaire, from Mansoura University. The pilot testing of the assessment tools was conducted according to the persons completing them for clarity and relevance.

#### The participant recruitment phase

Recruitment of participants. Among female students from Mansoura University, representatives from the faculties of Science, Agriculture, Education, and Arts were selected to obtain a representative sample from both theoretical and practical academic streams, which was a criterion for inclusion. Participants were eligible if they were female students aged 18–24 years old, enrolled in either grade 1–4. Students who met the inclusion criteria were approached in their respective faculties, where they obtained detailed information on the purpose of the study, procedures involved in the study, and measures taken to protect their confidentiality. All study participants had to provide written informed consent before participation.

### Data collection

- Data were collected through an online survey that was disseminated over WhatsApp via Google Forms to make data collection easy and accessible for the participants. The questionnaire included several parts, ranging in topic from the fast-food of a subject to identity formation:
- Part I: Socio-demographic data were obtained at baseline. Participants self-reported ages and body mass index (BMI, calculated from self-reported height and weight), parents’ educational attainment and occupation, family income, interest in health, and weight commitment.
- Part II: Eating habits qualitative Semi-structured interview guide: open-ended questions regarding participants’ dining practices, including frequency, timing, money spent, social context, and preferences. Participants were encouraged to elaborate on experiences and motivations. Part III: Fast-food consumption knowledge, which includes 14 items on the knowledge of fast food, its side effects, and principles of healthy nutrition.
- Part IV: Attitudes toward fast-food consumption, composed of 28 items regarding satisfaction, convenience, and perceived health implications.
- Part V: Perceived behavioral control (18 items)-assess the degree of perceived control over fast food consumption behavior.
- Part VI: Subjective norms (8 items), assessing peer, family, and societal influences on consumption decisions.
- Part VII: behavioral intention, was assessed using 8 items, referring to the intention of consuming fast foods in the future.

### Validity and reliability of the instruments

The tools used were adapted and validated within the framework designed by Didarloo et al. (2022). An Expert review by five specialists in community health and nursing education was conducted, after which the relevance of content and linguistic clarity of the scales were established for validity. Construct validity was measured by factor analysis, in which all items loaded well on their respective constructs. Reliability testing generated Cronbach’s alpha coefficients that fell between 0.81 and 0.93 for the subscales, indicating high internal consistency. In total, the whole survey took about 30 to 45 min. Data was anonymized and safely stored to ensure participant confidentiality. 

### Ethical considerations

The study was conducted in accordance with accepted ethical standards. The objectives, procedures, and possible risks of the study were clearly explained to the participants before giving their consent. Participation was entirely voluntary; withdrawal at any stage was allowed without any penalty. Data from participants who were withdrawn were not included in the analysis. All data collected from each participant were confidential, securely stored, and accessible only to the research team members. The study was conducted in full compliance with the ethical guidelines set by the IRB of Mansoura University.

### Data analysis

Data analysis was performed using IBM SPSS version 28.0, Armonk, NY, USA. Descriptive statistics in the form of mean, standard deviation, frequency, and percentage summarized participants’ sociodemographic data and their responses to TPB constructs of knowledge, attitude, perceived behavioral control, subjective norms, and behavioral intention. Data normality was checked using the Kolmogorov–Smirnov test, and indices of skewness and kurtosis. According to the distribution, either parametric or non-parametric tests were conducted. Independent-samples t-tests examined differences, whereas in one-way ANOVA with Tukey’s post-hoc test, TPB construct means were compared across more than two categories: for instance, categories of BMI, education, and income. In cases where assumptions of normality were violated, the Kruskal–Wallis H test was employed. Pearson’s correlation coefficient assessed the association among TPB constructs. A p-value less than 0.05 was considered statistically significant. In the psychometric evaluation, the internal consistency was evaluated by Cronbach’s alpha (α ≥ 0.70 is considered acceptable), while the test-retest reliability over the two-week interval was analyzed using Pearson’s r. The construct validity was determined by Exploratory Factor Analysis (EFA) and verified with Kaiser-Meyer-Olkin (KMO) and Bartlett’s tests. The findings were tabulated in detail with statistical values including t, F, H, r, and their respective significance levels for transparency.

## Results

Table 1 presents the characteristics of socio-demographic of the study participants. The mean age of participants was 21.71 ± 1.53 years, from 19 to 25 years. The average BMI was 26.96 ± 5.61. Participants were classified according to WHO adult BMI standards: 18.5–24.9 kg/m² as healthy weight (44.2%), 25.0–29.9 kg/m² as overweight (30.6%), and ≥ 30.0 kg/m² as obese (25.2%). Regarding parental education, 80.5% of participants’ parents had completed college education, 11.4% had secondary or lower education, and 8.1% held postgraduate degrees. Most parents were employed (86.5%), and the predominant family income level was medium (70.1%), followed by low (16.4%) and high (13.5%). A majority of participants reported interest in health (75.1%) and body weight management (96.6%). These findings demonstrate that the sample represented a relatively homogeneous group of young, educated women with moderate socioeconomic backgrounds, which provided a suitable context for examining behaviors of fast-food consumption.

**Table 1:** Sociodemographic characteristics of study participants

**Variable**	**Parameter**	**Statistics**	**Test**	***p*** **-value**
Age	**Mean ± SD**	21.71 ± 1.53	116.5	< 0.001
	**Min-max**	19–25		
BMI	**Mean ± SD**	26.96 ± 5.61	22.01	< 0.001
	**Min-max**	19–42		
	**Healthy weight**	170 (44.2%)		
	**Overweight**	118 (30.6%)		
	**Obese**	97 (25.2%)		
Parents’ education	**Secondary or lower**	44 (11.4%)	386.4	< 0.001
	**College**	310 (80.5%)		
	**Master or higher**	31 (8.1%)		
Parents’ work	**Non-employed**	52 (13.5%)	205.09	< 0.001
	**Employed**	333 (86.5%)		
Family income	**Low**	63 (16.4%)	235.04	< 0.001
	**Medium**	270 (70.1%)		
	**High**	52 (13.5%)		
Interest in health	**Yes**	289 (75.1%)	96.75	< 0.001
	**No**	96 (24.9%)		
Interest in body weight management	**Yes**	372 (96.6%)	334.75	< 0.001
	**No**	13 (3.4%)		

Data show means with SD for continuous variables andpercentages for categorical ones; all tests were significant at *p* < 0.001

Table 2 shows the knowledge scores of participants about fast food and its related issues. The mean total knowledge score was 5.37 ± 1.74 (range 1–11), a moderate level of knowledge. More than half (55.1%) knew that fast food has side effects, while 60.8% did not appreciate the value of healthy food choices. Over three-quarters (76.4%) indicated that health problems related to fast food did not occur immediately; however, only 41.8% of participants realized the importance of good nutrition for cardiovascular and chronic disease prevention, while 87.5% said that limiting fast food was a part of a healthy lifestyle. Finally, 80.3% were unaware of the use of preservatives in fast food ingredients. Overall, these findings suggest that participants have a good general understanding of the health consequences of fast food; however, when it comes to nutrition and environmental issues, their understanding was more limited.

**Table 2:** Knowledge score among participants

**Variable**	**Parameter**	**Statistics**
Do fast foods have any side effects for the body	**No**	212 (55.1%)
	**Yes**	173 (44.9%)
Is careful control of eating and eating healthy foods important	**No**	234 (60.8%)
	**Yes**	151 (39.2%)
If we consume a lot of fast food, the diseases caused by consuming them usually appear on the same day	**No**	294 (76.4%)
	**Yes**	91 (23.6%)
Does proper and healthy nutrition play a role in the incidence of chronic diseases?	**No**	224 (58.2%)
	**Yes**	161 (41.8%)
Do we have to limit the consumption of fast foods to have a healthy life?	**No**	48 (12.5%)
	**Yes**	337 (87.5%)
Does the constant use of fast food in the restaurant environment cause you to stay away from the family environment?	**No**	310 (80.5%)
	**Yes**	75 (19.5%)
Does the packaging of fast food (use of plastic materials) have an impact on the environment?	**No**	273 (70.9%)
	**Yes**	112 (29.1%)
Is the cause of obesity in most children, adolescents and young people today, especially in developing countries, eating unhealthy foods such as fast food?	**No**	233 (60.5%)
	**Yes**	152 (39.5%)
Is salt, oil and sugar used properly in the preparation of fast foods?	**No**	306 (79.5%)
	**Yes**	79 (20.5%)
By consuming fast food in every meal, is the body’s need for the food groups needed by the body fully met?	**No**	173 (44.9%)
	**Yes**	212 (55.1%)
Do fast food restaurants change the oil used for each fast-food preparation?	**No**	190 (49.4%)
	**Yes**	195 (50.6%)
Are the ingredients used in the preparation of fast foods instead of meat, such as sausages, useful for the body?	**No**	284 (73.8%)
	**Yes**	101 (26.2%)
Does regular consumption of fast food, especially in children, increase the risk of diseases other than obesity and nutritional problems such as allergies and asthma?	**No**	232 (60.3%)
	**Yes**	153 (39.7%)
Are preservatives used in raw materials for fast food (including sausages)?	**No**	309 (80.3%)
	**Yes**	76 (19.7%)
Total score	**Mean ± SD**	5.37 ± 1.74
	**Min-max**	1–11

Knowledge scores range from1 to 11, reflecting participants' understanding of fast food’s health and environmental impacts

Table 3 describes participants’ attitudes about fast food. The total mean attitude score was 92.63 ± 15.06 (range 66–123), indicating mostly positive attitudes toward fast food, even though participants were aware of the health consequences of fast food. For example, 67.8% of the participants agreed that fast food is delicious, while 57% agreed that fast food leads to weight gain, and 68.3% agreed that fast food promotes inactivity. Additionally, 44.7% of the participants agreed that fast food saves time. Socially, 53.5% agreed that eating fast food frequently reduced family meals, while 52.7% of participants agreed that fast food is or can be habitual. In summary, the attitudes of the participants revealed an internal struggle between convenience-driven preferences and healthy beliefs.

**Table 3:** Attitude score among participants

**Variable**	**Parameter**	**Statistics**
Fast food is delicious	**Strongly disagree**	16 (4.2%)
	**Disagree**	52 (13.5%)
	**Do not know**	56 (14.5%)
	**Agree**	100 (26%)
	**Strongly agree**	161 (41.8%)
Eat delicious food for me	**Extremely unimportant**	74 (19.2%)
	**Unimportant**	73 (19%)
	**I have no idea**	68 (17.7%)
	**Important**	106 (27.5%)
	**Extremely important**	64 (16.6%)
I feel satisfied after eating fast food.	**Strongly disagree**	64 (16.6%)
	**Disagree**	72 (18.7%)
	**Do not know**	40 (10.4%)
	**Agree**	79 (20.5%)
	**Strongly agree**	130 (33.8%)
Feeling satisfied after eating for me	**Extremely unimportant**	71 (18.4%)
	**Unimportant**	66 (17.1%)
	**I have no idea**	49 (12.7%)
	**Important**	40 (10.4%)
	**Extremely important**	159 (41.3%)
Eating fast food (3 or more times a week) is good for health.	**Strongly disagree**	127 (33%)
	**Disagree**	96 (24.9%)
	**Do not know**	141 (36.6%)
	**Agree**	16 (4.2%)
	**Strongly agree**	5 (1.3%)
Maintaining the health of the body for me	**Extremely unimportant**	16 (4.2%)
	**Unimportant**	21 (5.5%)
	**I have no idea**	25 (6.5%)
	**Important**	49 (12.7%)
	**Extremely important**	274 (71.2%)
I think eating fast food (3 or more times a week) probably causes weight gain.	**Strongly disagree**	12 (3.1%)
	**Disagree**	16 (4.2%)
	**Do not know**	80 (20.8%)
	**Agree**	93 (24.2%)
	**Strongly agree**	184 (47.8%)
Keeping the weight in a normal size for me.	**Extremely unimportant**	12 (3.1%)
	**Unimportant**	52 (13.5%)
	**I have no idea**	59 (15.3%)
	**Important**	71 (18.4%)
	**Extremely important**	191 (49.6%)
Eating fast food (3 times or more a week) is very convenient for me.	**Strongly disagree**	143 (37.1%)
	**Disagree**	71 (18.4%)
	**Do not know**	47 (12.2%)
	**Agree**	37 (9.6%)
	**Strongly agree**	87 (22.6%)
Having a comfortable food source for me	**Extremely unimportant**	55 (14.3%)
	**Unimportant**	34 (8.8%)
	**I have no idea**	40 (10.4%)
	**Important**	39 (10.1%)
	**Extremely important**	217 (56.4%)
Eating fast food (3 or more times a week) reduces the amount of work I have to do to prepare food (for example, planning, preparing and cleaning).	**Strongly disagree**	128 (33.2%)
	**Disagree**	41 (10.6%)
	**Do not know**	66 (17.1%)
	**Agree**	48 (12.5%)
	**Strongly agree**	102 (26.5%)
Reducing the amount of work I have to do to prepare food (for example, planning, preparing and cleaning) for me	**Extremely unimportant**	130 (33.8%)
	**Unimportant**	89 (23.1%)
	**I have no idea**	32 (8.3%)
	**Important**	9 (2.3%)
	**Extremely important**	125 (32.5%)
Eating fast food (3 or more times a week) saves me time.	**Strongly disagree**	33 (8.6%)
	**Disagree**	56 (14.5%)
	**Do not know**	87 (22.6%)
	**Agree**	37 (9.6%)
	**Strongly agree**	172 (44.7%)
Saving time spent preparing food for me …	**Extremely unimportant**	29 (7.5%)
	**Unimportant**	27 (7%)
	**I have no idea**	148 (38.4%)
	**Important**	36 (9.4%)
	**Extremely important**	145 (37.7%)
Eating fast food gives me the opportunity to eat my food wherever I want (for example in a restaurant, car and home).	**Strongly disagree**	91 (23.6%)
	**Disagree**	78 (20.3%)
	**Do not know**	44 (11.4%)
	**Agree**	40 (10.4%)
	**Strongly agree**	132 (34.3%)
To be able to eat wherever I want ….	**Extremely unimportant**	91 (23.6%)
	**Unimportant**	78 (20.3%)
	**I have no idea**	44 (11.4%)
	**Important**	40 (10.4%)
	**Extremely important**	132 (34.3%)
Eating fast food (3 or more times a week) is relatively cheap compared to restaurant food.	**Strongly disagree**	58 (15.1%)
	**Disagree**	121 (31.4%)
	**Do not know**	72 (18.7%)
	**Agree**	60 (15.6%)
	**Strongly agree**	74 (19.2%)
Cheap food for me ……	**Extremely unimportant**	70 (18.2%)
	**Unimportant**	6 (1.6%)
	**I have no idea**	74 (19.2%)
	**Important**	139 (36.1%)
	**Extremely important**	96 (24.9%)
Eating (3 or more times a week) in fast food restaurants allows me to get out of the house.	**Strongly disagree**	46 (11.9%)
	**Disagree**	54 (14%)
	**Do not know**	127 (33%)
	**Agree**	113 (29.4%)
	**Strongly agree**	45 (11.7%)
Leaving home for any reason for me …	**Extremely unimportant**	47 (12.2%)
	**Unimportant**	51 (13.2%)
	**I have no idea**	121 (31.4%)
	**Important**	115 (29.9%)
	**Extremely important**	51 (13.2%)
Eating fast food (3 or more times a week) reduces the possibility of eating traditional foods with the family	**Strongly disagree**	90 (23.4%)
	**Disagree**	51 (13.2%)
	**Do not know**	38 (9.9%)
	**Agree**	129 (33.5%)
	**Strongly agree**	77 (20%)
Maintain opportunities to eat traditional food with my family …	**Extremely unimportant**	88 (22.9%)
	**Unimportant**	25 (6.5%)
	**I have no idea**	55 (14.3%)
	**Important**	92 (23.9%)
	**Extremely important**	125 (32.5%)
Eating fast food (3 or more times a week) causes habituation.	**Strongly disagree**	100 (26%)
	**Disagree**	47 (12.2%)
	**Do not know**	74 (19.2%)
	**Agree**	71 (18.4%)
	**Strongly agree**	93 (24.2%)
Constant consumption of fast food for me …	**Extremely unimportant**	72 (18.7%)
	**Unimportant**	53 (13.8%)
	**I have no idea**	77 (20%)
	**Important**	131 (34%)
	**Extremely important**	52 (13.5%)
Eating fast food (3 times or more per week) causes inactivity.	**Strongly disagree**	31 (8.1%)
	**Disagree**	33 (8.6%)
	**Do not know**	58 (15.1%)
	**Agree**	126 (32.7%)
	**Strongly agree**	137 (35.6%)
A sedentary and sedentary life for my health.	**Extremely unimportant**	143 (37.1%)
	**Unimportant**	32 (8.3%)
	**I have no idea**	36 (9.4%)
	**Important**	86 (22.3%)
	**Extremely important**	88 (22.9%)
Eating fast food (3 or more times a week) is harmful to the environment.	**Strongly disagree**	68 (17.7%)
	**Disagree**	66 (17.1%)
	**Do not know**	44 (11.4%)
	**Agree**	56 (14.5%)
	**Strongly agree**	151 (39.2%)
Environmental protection for me ………	**Extremely unimportant**	86 (22.3%)
	**Unimportant**	62 (16.1%)
	**I have no idea**	43 (11.2%)
	**Important**	55 (14.3%)
	**Extremely important**	139 (36.1%)
Total score	**Mean ± SD**	92.63 ± 15.06
	**Min-max**	66–123

Attitude scores (66–123) reflect positive views toward fast food, despite health awareness

Table 4 shows the results for the perceived behavioral control regarding eating fast foods. The mean for perceived behavioral control was 58.39 ± 9.47, indicating a moderate level of self-regulation. Most of the participants thought that a lack of free time got in the way of eating healthy (62.1%), and 49.1% thought that time was what led to increasing fast food consumption. Emotional and social triggers were significant; 77.4% admitted cravings prompted them to eat fast foods more, and 66.8% reported poor cooking skills. These data suggest that even if college students are aware and trying to eat healthily, their perceived behavioral control is limited by environmental and lifestyle habits that most college students encounter.

**Table 4:** Perceived behavioral control score among participants

**Variable**	**Parameter**	**Statistics**
My lifestyle is such that I will have little free time next month.	**Strongly disagree**	29 (7.5%)
	**Disagree**	67 (17.4%)
	**Do not know**	50 (13%)
	**Agree**	93 (24.2%)
	**Strongly agree**	146 (37.9%)
Having less free time causes me to eat fast food 3 or more times a week.	**Strongly disagree**	70 (18.2%)
	**Disagree**	66 (17.1%)
	**Do not know**	60 (15.6%)
	**Agree**	98 (25.5%)
	**Strongly agree**	91 (23.6%)
I feel guilty about eating certain foods	**Strongly disagree**	121 (31.4%)
	**Disagree**	66 (17.1%)
	**Do not know**	37 (9.6%)
	**Agree**	66 (17.1%)
	**Strongly agree**	95 (24.7%)
Feeling guilty after eating fast food prevents me from eating fast food (3 times or more a week).	**Strongly disagree**	116 (30.1%)
	**Disagree**	43 (11.2%)
	**Do not know**	36 (9.4%)
	**Agree**	31 (8.1%)
	**Strongly agree**	159 (41.3%)
Sometimes I want to eat some food.	**Strongly disagree**	53 (13.8%)
	**Disagree**	53 (13.8%)
	**Do not know**	92 (23.9%)
	**Agree**	187 (48.6%)
	**Strongly agree**	187 (48.6%)
My desire to eat fast food makes me eat fast food often (3 times or more a week).	**Strongly disagree**	43 (11.2%)
	**Disagree**	20 (5.2%)
	**Do not know**	24 (6.2%)
	**Agree**	44 (11.4%)
	**Strongly agree**	254 (66%)
Most of the time I only eat	**Strongly disagree**	130 (33.8%)
	**Disagree**	8 (2.1%)
	**Do not know**	44 (11.4%)
	**Agree**	65 (16.9%)
	**Strongly agree**	138 (35.8%)
Eating alone allows me to eat fast food frequently (3 times or more a week).	**Strongly disagree**	9 (2.3%)
	**Disagree**	26 (6.8%)
	**Do not know**	39 (10.1%)
	**Agree**	66 (17.1%)
	**Strongly agree**	245 (63.6%)
I cannot prepare food for myself, because I do not know how to cook.	**Strongly disagree**	133 (34.5%)
	**Disagree**	65 (16.9%)
	**Do not know**	45 (11.7%)
	**Agree**	36 (9.4%)
	**Strongly agree**	106 (27.5%)
Because I do not know how to cook, I prefer to eat fast food frequently (3 times or more a week).	**Strongly disagree**	80 (20.8%)
	**Disagree**	8 (2.1%)
	**Do not know**	40 (10.4%)
	**Agree**	40 (10.4%)
	**Strongly agree**	217 (56.4%)
I have to be careful about the foods I eat because of my weight.	**Strongly disagree**	181 (47%)
	**Disagree**	37 (9.6%)
	**Do not know**	57 (14.8%)
	**Agree**	39 (10.1%)
	**Strongly agree**	71 (18.4%)
Worrying about my weight prevents me from eating fast food (3 times or more a week).	**Strongly disagree**	154 (40%)
	**Disagree**	83 (21.6%)
	**Do not know**	26 (6.8%)
	**Agree**	8 (2.1%)
	**Strongly agree**	114 (29.6%)
I have to take care of what I eat for the sake of my health.	**Strongly disagree**	61 (15.8%)
	**Disagree**	52 (13.5%)
	**Do not know**	79 (20.5%)
	**Agree**	36 (9.4%)
	**Strongly agree**	157 (40.8%)
Concerns about my health keep me from eating fast food (often or more than once a week).	**Strongly disagree**	133 (34.5%)
	**Disagree**	26 (6.8%)
	**Do not know**	137 (35.6%)
	**Agree**	20 (5.2%)
	**Strongly agree**	69 (17.9%)
I have no access to fast food where I live.	**Strongly disagree**	91 (23.6%)
	**Disagree**	78 (20.3%)
	**Do not know**	44 (11.4%)
	**Agree**	40 (10.4%)
	**Strongly agree**	132 (34.3%)
Lack of access to fast food, causes me not to eat fast food frequently (3 times or more per week).	**Strongly disagree**	91 (23.6%)
	**Disagree**	78 (20.3%)
	**Do not know**	44 (11.4%)
	**Agree**	40 (10.4%)
	**Strongly agree**	132 (34.3%)
How much I eat fast food next month is more up to me.	**Strongly disagree**	80 (20.8%)
	**Disagree**	121 (31.4%)
	**Do not know**	72 (18.7%)
	**Agree**	51 (13.2%)
	**Strongly agree**	61 (15.8%)
From next month, I can reduce the number of times I eat my fast food	**Strongly disagree**	79 (20.5%)
	**Disagree**	3 (0.8%)
	**Do not know**	50 (13%)
	**Agree**	128 (33.2%)
	**Strongly agree**	125 (32.5%)
Total score	**Mean ± SD**	58.39 ± 9.47
	**Min-max**	42–81

Perceived behavioral control scores (42–81) show moderate self-regulation in eating fast food

Table 5 provides information on subjective norms and their influence on dietary patterns. The mean score for subjective norms was 25.46 ± 5.54 (range 12–40), indicating a moderate level of social influence. Family attitudes exerted the greatest impact: 74.3% of participants reported that family attitudes about nutrition were important, and 51.7% stated that their family encouraged the consumption of fast foods three or more times per week. In contrast, peers had less influence, with only 34% indicating that friends’ perspectives were significant. This suggests that family is the primary social factor influencing dietary behaviors, outweighing peer or professional opinions.

**Table 5:** Subjective norms among participants

**Variable**	**Parameter**	**Statistics**
The people I respect by eating my fast food are.	**Strongly disagree**	77 (20%)
	**Disagree**	39 (10.1%)
	**Do not know**	110 (28.6%)
	**Agree**	68 (17.7%)
	**Strongly agree**	91 (23.6%)
People’s opinion about eating fast food, for me.	**Extremely unimportant**	44 (11.4%)
	**Unimportant**	46 (11.9%)
	**I have no idea**	110 (28.6%)
	**Important**	106 (27.5%)
	**Extremely important**	79 (20.5%)
In general, my family members think that I should eat fast food 3 or more times a week.	**Strongly disagree**	123 (31.9%)
	**Disagree**	34 (8.8%)
	**Do not know**	29 (7.5%)
	**Agree**	104 (27%)
	**Strongly agree**	95 (24.7%)
My family members’ opinion about my nutrition, for me.	**Extremely unimportant**	25 (6.5%)
	**Unimportant**	25 (6.5%)
	**I have no idea**	49 (12.7%)
	**Important**	79 (20.5%)
	**Extremely important**	207 (53.8%)
In general, my friends think I should eat fast food 3 or more times a week.	**Strongly disagree**	186 (48.3%)
	**Disagree**	43 (11.2%)
	**Do not know**	63 (16.4%)
	**Agree**	45 (11.7%)
	**Strongly agree**	48 (12.5%)
My friends’ opinion about my nutrition, for me	**Extremely unimportant**	72 (18.7%)
	**Unimportant**	53 (13.8%)
	**I have no idea**	77 (20%)
	**Important**	131 (34%)
	**Extremely important**	52 (13.5%)
Health experts think I should eat fast food 3 or more times a week	**Strongly disagree**	45 (11.7%)
	**Disagree**	32 (8.3%)
	**Do not know**	54 (14%)
	**Agree**	122 (31.7%)
	**Strongly agree**	132 (34.3%)
The opinion of health professionals about my nutrition, for me …	**Extremely unimportant**	153 (39.7%)
	**Unimportant**	31 (8.1%)
	**I have no idea**	33 (8.6%)
	**Important**	82 (21.3%)
	**Extremely important**	86 (22.3%)
Total score	**Mean ± SD**	25.46 ± 5.54
	**Min-max**	12–40

Subjective norms scores (12–40) show moderate influence, with family being the main factor

The findings regarding behavioral intention are documented in Table 6, which indicates a very strong willingness to consume healthier foods. The mean score for behavioral intention was 30.22 ± 3.52 (for a scale from 20 to 38). The majority of participants (66.6%) indicated they planned to decrease the consumption of fast foods in the next month, while 70.6% indicated that they planned to increase the consumption of healthier foods. However, only 32.7% of participants said they planned to visit a nutritionist on a regular basis. These findings indicate that although the intention to eat better was quite high, seeking out professional nutritional advice was rarely done.

**Table 6:** Behavioral intention assessment among participants

**Variable**	**Parameter**	**Statistics**
I plan to eat less fast-food next month.	**Strongly disagree**	20 (5.2%)
	**Disagree**	67 (17.4%)
	**Do not know**	40 (10.4%)
	**Agree**	83 (21.6%)
	**Strongly agree**	175 (45.5%)
I plan to use the right foods for my health in my diet in the coming weeks.	**Strongly disagree**	12 (3.1%)
	**Disagree**	49 (12.7%)
	**Do not know**	52 (13.5%)
	**Agree**	71 (18.4%)
	**Strongly agree**	201 (52.2%)
I will try to pay attention to the type of food I consume next month.	**Strongly disagree**	43 (11.2%)
	**Disagree**	74 (19.2%)
	**Do not know**	33 (8.6%)
	**Agree**	6 (1.6%)
	**Strongly agree**	229 (59.5%)
Next month, I plan to pay attention to the type of food that I pay for each meal and how it is prepared, that is, healthy foods.	**Strongly disagree**	19 (4.9%)
	**Disagree**	20 (5.2%)
	**Do not know**	31 (8.1%)
	**Agree**	31 (8.1%)
	**Strongly agree**	284 (73.8%)
I will try to pay attention to my diet in future trips and parties.	**Strongly disagree**	128 (33.2%)
	**Disagree**	41 (10.6%)
	**Do not know**	66 (17.1%)
	**Agree**	48 (12.5%)
	**Strongly agree**	102 (26.5%)
I plan to see a nutritionist at least once every 6 months for my diet in the coming months.	**Strongly disagree**	124 (32.2%)
	**Disagree**	83 (21.6%)
	**Do not know**	52 (13.5%)
	**Agree**	8 (2.1%)
	**Strongly agree**	118 (30.6%)
I plan to stop eating fast food next month.	**Strongly disagree**	10 (2.6%)
	**Disagree**	38 (9.9%)
	**Do not know**	55 (14.3%)
	**Agree**	29 (7.5%)
	**Strongly agree**	253 (65.7%)
I will try to eat more home diet next month.	**Strongly disagree**	14 (3.6%)
	**Disagree**	16 (4.2%)
	**Do not know**	79 (20.5%)
	**Agree**	30 (7.8%)
	**Strongly agree**	246 (63.9%)
Total score	**Mean ± SD**	30.22 ± 3.52
	**Min-max**	20–38

Behavioral intention scores (20–38) show strong intention to reduce fast food and adopt healthier habits

In Table 7, mean scores for the five TPB constructs were compared based on participants’ demographics (BMI, parental education level, parental occupation, and household income). Significant differences were found across BMI categories for all variables (*p* < 0.05), with obese participants reporting the greatest scores for knowledge, attitude, perceived control, subjective norms, and behavioral intention. There were no significant differences across parental education categories; however, there were significant differences in knowledge and attitude between participants with employed vs. unemployed parents. For household income, there were significant differences across all constructs, with participants reporting higher income exhibiting greater knowledge and intention to behave. These findings suggest that socioeconomic status and family context are important influences on dietary cognition and behavior.

**Table 7:** Associations of knowledge, attitude, perceived behaviour, subjective norms, and behavioural intention assessment scores according to characteristics of participants

**Variable**	**Parameter**	**Knowledge score**	**Attitude score**	**Perceived Behavioral Control score**	**Subjective norms score**	**Behavioral Intention Assessment score**
BMI	**Healthy weight (** ***n*** ** = 170)**	5.28 ± 1.74	82.71 ± 7.39	52.76 ± 6.86	21.81 ± 4.55	30.38 ± 3.42
	**Overweight (** ***n*** ** = 118)**	5.86 ± 1.62	89.45 ± 10.27	57.39 ± 7.15	26.15 ± 3.39	28.33 ± 3.06
	**Obese (** ***n*** ** = 97)**	4.94 ± 1.77	113.9 ± 6.2	69.48 ± 5.63	31.03 ± 4.13	32.24 ± 3.02
*p*-value		**< 0.001**	**< 0.001**	**< 0.001**	**< 0.001**	**< 0.001**
Parents’ education	**Secondary or lower (** ***n*** ** = 44)**	5.5 ± 1.77	90.7 ± 13.33	58.66 ± 9.34	25.02 ± 4.44	30.30 ± 3.43
	**College (** ***n*** ** = 310)**	5.35 ± 1.76	93.25 ± 15.06	58.44 ± 9.43	25.71 ± 5.58	30.15 ± 3.55
	**Master’s degree or higher (** ***n*** ** = 31)**	5.35 ± 1.64	89.23 ± 16.98	57.52 ± 10.29	23.65 ± 6.31	30.77 ± 3.45
*P*-value		**0.87**	**0.24**	**0.85**	**0.12**	**0.63**
Parents’ occupation	**Non-employed (** ***n*** ** = 52)**	4.88 ± 1.72	96.67 ± 14.26	59.92 ± 10.70	26.02 ± 4.88	29.50 ± 3.45
	**Employed (** ***n*** ** = 333)**	5.45 ± 1.74	92 ± 15.103	58.15 ± 9.26	25.38 ± 5.64	30.33 ± 3.52
*p*-value		**0.031**	**0.037**	**0.21**	**0.43**	**0.11**
Family income	**Low (** ***n*** ** = 63)**	5.7 ± 1.7	84.37 ± 5.67	52.83 ± 5.24	24.08 ± 4.07	29 ± 4.11
	**Medium (** ***n*** ** = 270)**	5.44 ± 1.73	89.9 ± 13.3	57.76 ± 9.35	24.46 ± 5.12	29.89 ± 3.19
	**High (** ***n*** ** = 52)**	4.6 ± 1.7	116.87 ± 3.51	68.42 ± 6.12	32.35 ± 4.17	33.38 ± 2.56
*p*-value		**0.001**	**< 0.001**	**< 0.001**	**< 0.001**	**< 0.001**

Differences in TPB constructs are significant across BMI and income (*p* < 0.05)

Table 8 presents the correlation matrix among TPB constructs. Knowledge negatively correlated weakly with behavioral intention (*r* = − 0.181, *p* < 0.001), whereas attitude (*r* = 0.331, *p* < 0.001), perceived behavioral control (*r* = 0.202, *p* < 0.001), and subjective norms (*r* = 0.253, *p* < 0.001) were moderately correlated positively with behavioral intention. Attitude and perceived behavioral control were most strongly related to one another (*r* = 0.826, *p* < 0.001). These findings are consistent with the Theory of Planned Behavior, in that attitude, perceived control, and subjective norms are all significant predictors of intention to eat more healthily.

**Table 8:** Correlations between knowledge, attitude, perceived behaviour, subjective norms, and behavioural intention assessment scores

		Knowledge score	Attitude score	Perceived Behavioral Control score	Subjective norms score
Attitude score	***r*** **-value**	-0.091			
	***p*** **-value**	0.073			
Perceived Behavioural Control score	***r*** **-value**	0.130^*^	0.826^**^		
	***p*** **-value**	0.011	< 0.001		
Subjective norms score	***r*** **-value**	-0.065	0.736^**^	0.461^**^	
	***p*** **-value**	0.204	< 0.001	< 0.001	
Behavioral Intention Assessment score	***r*** **-value**	-0.181^**^	0.331^**^	0.202^**^	0.253^**^
	***p*** **-value**	< 0.001	< 0.001	< 0.001	< 0.001

*. Correlation is significant at the 0.05 level (2-tailed)

**. Correlation is significant at the 0.01 level (2-tailed)

Correlations show attitude, perceived control, and subjective normspositively predict intention, while knowledge has a weak negative correlation

## Discussion

Most of the previous literature indicates that fast-food consumption among female university students has been increasing during the past three decades [2]. The rapid expansion of fast-food restaurants globally, which is supported by factors such as a lack of time, accessibility of restaurants, speedy service, and easy availability, has transformed fast food into an essential component of many diets today [3]. This research tries to explore the relationship between fast-food consumption and identity formation for 385 female university students by questioning how their eating reflects and constructs their sense of selves. The mean age of the participants in the present study was 21.71 ± 1.53 years. This is in agreement with a previous study done by Hassan and El Magrabi in 2021, which reported an average age of 20.97 ± 1.14 among Egyptian university students; the age range was also 18–25 years [17]. Similarly, El-Gilany and Elkhawaga, in 2012, conducted a study in the El-Mansoura governorate, finding a mean age of 20.2 ± 1.7 years within the same age range [18]. These agreements suggest that the ages of university students tend to cluster in the mentioned range, which can be explained by standardized ages for undergraduate studies. These findings indicate greater homogeneity because the students stay within the age brackets due to the fact that graduation usually happens at the conventional times. Regarding the socioeconomic class, the highest percentage of the participants in the present study reported medium family income levels (70.1%), followed by low (16.4%) and high (13.5%) income levels. Moreover, 86.5% of participants had employed parents. This finding is in accordance with Hassan and El Magrabi (2021), who reported that middle socioeconomic status was the major category in their sample too [17]. However, this finding contradicts that of El-Gilany and Elkhawaga (2012), who reported that two-thirds of university students belonged to the high socioeconomic class [18]. This may be because of differences in the method of sampling or due to regional economic variations [19, 20]. Regarding knowledge about fast food, the mean total knowledge score of participants in the present study was 5.37 ± 1.74, indicating moderate knowledge. In agreement with this, Hassan and El Magrabi (2021) stated that 40% of their sample had fair knowledge scores, and about one-third had poor knowledge levels [17]. Abraham et al. (2016) established that university students have reasonable knowledge regarding nutrition and health, thus supporting the present results [21]. Recent evidence also underscores that nutritional knowledge among the youth is superficial; media and peers influence misunderstandings about the healthiness of fast foods [1]. Similar international findings have echoed these gaps, showing limited awareness of food ingredients, additives, and preservatives among students in Nepal, Korea, Japan, and Iran [22–24]. A particularly notable finding of this study was that 80.3% of participants were unaware of the use of preservatives in fast-food ingredients. This reflects a substantial gap in nutritional literacy among female university students, particularly regarding food additives and processing methods. Similar trends of limited knowledge about food composition and labeling have been reported among young adults in Bangladesh, Iran, and Portugal [4, 5]. Studies conducted in Egypt and neighboring countries have also confirmed that university students often associate fast food with convenience and taste rather than its chemical content or long-term health effects [16]. Evidence from behavioral studies further suggests that low awareness of food processing is linked with higher fast-food consumption and reduced health-oriented decision making [25, 26]. This unawareness may increase the risk of exposure to harmful additives and contribute to unhealthy consumption patterns over time. The finding underscores the need for nurse-led educational interventions that emphasize food-label interpretation, awareness of preservatives and additives, and the broader health implications of highly processed diets. Strengthening such awareness within university settings could play a crucial role in promoting informed food choices and reducing health risks associated with fast-food consumption. This study revealed that participants’ mean attitude score towards fast-food consumption was 92.63 ± 15.06, indicating a high attitude score. This is supported by Hassan and El Magrabi, who reported in 2021 that more than half of their study participants had favorable attitudes towards fast foods [17]. The studies by Hu et al. in 2016 [20] and Akhter in 2019 [27] also attested to positive attitudes among university students. In addition, 67.8% of participants agreed or strongly agreed that fast food is delicious, highlighting its strong sensory appeal. Behavioral research demonstrates that attitudes toward fast-food consumption are shaped by social norms, perceived behavioral control, and subjective norms, as explained by the Theory of Planned Behavior in various youth populations [23, 24, 28–31]. Similar findings were also reported among Iranian university students, where the Theory of Planned Behavior significantly predicted fast-food consumption intentions and behaviors [32]. 

Moreover, 41.3% of participants reported respecting the opinions of others who consumed fast food, reflecting the potential influence of peer norms on fast-food consumption behavior among female university students and suggesting that social approval may reinforce dietary choices. Such similarities may reflect the strong worldly appeal of fast foods due to convenience, taste, and social influences and time scarcity in food choices [33]. However, Pelletier et al. reported in 2013 that one-third of students in the USA showed low attitudes towards fast-food consumption, which was contrary to this result [22]. Correspondingly, cultural acceptance and social trends have remained key predictors of positive fast-food attitude among youth [4]. TPB-based studies across diverse cultural contexts also emphasize the central role of peer influence and normative beliefs in shaping fast-food consumption behaviors [23, 25]. 

In this present study, the mean BMI was 26.96 ± 5.61, and 30.6% were overweight, while 25.2% were obese. It aligns with the results of Alfawaz (2012), in which it has been reported that 25% of students were overweight or obese [23]. It is important to note that these findings confirm that unhealthy eating behaviors, such as fast-foodconsumption, are associated with higher BMI and adverse dietary patterns among university students [23, 34, 35]. Alfawaz also provided associations of fast-food consumption with poor self-rated health, weight dissatisfaction, and difficulties in the preparation of healthy meals, which match the current study. Recently, such findings were also confirmed in multi-country analyses that pointed to frequent fast-food consumption as one of the factors of BMI increase and metabolic risk in university women [9]. Additional behavioral studies have linked fast-food consumption with negative weight outcomes within TPB frameworks, reinforcing psychosocial drivers of unhealthy eating [26]. 

More than half of the participants in the present study acknowledged the adverse health implications of fast food. In this regard, Alfawaz, in 2012, reported that 85.8% of female students knew of the unhealthiness of fast foods [23]. Such agreement is expected because increased media and health awareness campaigns have focused on adverse health implications concerning fast food. Recent campaigns tend to increase awareness with little improvement in terms of changing consumption behaviors [14]. Moreover, convenience turned out to be a leading motivating factor for fast food consumption in the present research. This is in line with the studies by Ismail (2016) [35], which identified convenience as a major factor motivating university students to consume fast foods. In addition, previous evidence suggests that consumer responses to information on food additives may influence food choices and health-related decision-making [36]. Similar findings were also reported by Rui-Hui (2015) [37], emphasizing the role of convenience in fast-food consumption. These findings are further supported by behavioral studies highlighting the role of perceived convenience and environmental factors in shaping fast-food consumption patterns [38]. These findings highlight the need to address convenience-oriented marketing strategies in public health initiatives. Evidence from cross-national research indicates that affordability and delivery accessibility have aggravated this trend during the post-COVID era [6]. Behavioral literature further supports that perceived convenience and accessibility significantly predict fast-food consumption across student populations [26, 37]. Moreover, convenience turned out to be a leading motivating factor [38] for fast food consumption in the present research, which is in tune with the studies by Ismail (2016) [35] and Rui-Hui (2015) [37] that convenience was a major factor in motivating university students to consume fast foods. These findings highlight the need to address convenience-oriented marketing strategies in public health initiatives. Evidence from cross-national research indicates that affordability and delivery accessibility have aggravated this trend during the post-COVID era [6]. Behavioral literature further supports that perceived convenience and accessibility significantly predict fast-food consumption across student populations [25, 26].

### Strengths and limitations

This study addresses an underexplored area—fast-food consumption and identity formation among female university students in Egypt. The inclusion of participants from different faculties ensured diverse perspectives, while the use of validated tools enhanced the reliability of the findings. Beyond confirming existing evidence on convenience and affordability, the study identified distinct psychosocial factors—such as self-concept, peer influence, and social affiliation as major predictors of fast-food consumption. These insights extend previous research and offer new implications for nursing-led health education and behavioral interventions.

However, the study relied on self-reported data, which may have introduced recall or social desirability bias. Its cross-sectional design also limits causal inference. Furthermore, psychosocial factors such as stress or self-image were not explored in depth. Future longitudinal and mixed-method studies are recommended to validate and expand these findings.

## Conclusion

The research illustrated that convenience, socioeconomic status, and cultural factors strongly influenced the consumption of fast food. Although respondents possessed moderate knowledge of the health implications of fast food, their enjoyment of eating it and limited intent to change behavior indicate the need for tailored strategies. Consistent with existing literature, the role of family appeared as a dominant influence on consumption levels of fast food, emphasizing the significance of addressing family involvement in health education programming.

### Recommendations

- Develop targeted nutrition education programs for university students to focus on the health risks of fast food and strategies to consume healthy food in a time and budget-constrained environment.
- Develop family-oriented campaigns to educate parents about their role and influence in developing healthy eating patterns, and the importance of supporting healthy eating choices.
- Support policies that limit fast food entrepreneurs’ access to university campuses or promote healthy and affordable food options.
- Include behavioral strategies like motivational interviewing and goal setting; this can enhance intentions toward healthy eating behavior among students.
- Longitudinal studies should be conducted to better understand the causality among knowledge, attitudes, and behaviors, and the sample should be expanded to male students and other age groups.

## Supplementary Information

Supplementary Material 1.

Supplementary Material 2.

## Data Availability

The availability of all the data and materials needed to complete the inquiry is attested to by the corresponding author.

## Abbreviation

BMI: Body Mass Index

## Declarations

This study was conducted in accordance with theethical principles of the Declaration of Helsinki.

This study was conducted in accordance with the ethical principles of the Declaration of Helsinki.
