# Supplementary material for: Adult women identities on the menu: deconstructing fast-food consumption among university students
Source: BMC Nutr. 2026 May 5;12:87. doi: 10.1186/s40795-026-01287-3 (PMC13147893; doi:10.1186/s40795-026-01287-3)
Supplement: Supplementary file 1 — Supplementary Material 1. [file 40795_2026_1287_MOESM1_ESM.docx]

**Part I: Socio-demographic data of Participants:**

**Section A: Socio-demographic Characteristics**

1. **Age**
   - _____ years (please write your exact age)
2. **Height and Weight**
   - Height: _____ cm
   - Weight: _____ kg
     (BMI will be calculated by the researcher from these values.)
3. **Parents’ Level of Education**
   - ☐ Secondary or lower (e.g., high school or below)
   - ☐ College degree
   - ☐ Master’s degree or higher
4. **Parents’ Employment Status**
   - ☐ Both non-employed
   - ☐ At least one employed
5. **Family Monthly Income**
   - ☐ Low (below average standard of living)
   - ☐ Medium (average standard of living)
   - ☐ High (above average standard of living)

**Section B: Health and Lifestyle Characteristics**

1. **Are you interested in health-related topics (nutrition, exercise, disease prevention, etc.)?**
   - ☐ Yes
   - ☐ No
2. **Are you personally interested in managing or controlling your body weight?**
   - ☐ Yes
   - ☐ No

✅ Notes:

- Questions (1–2) produce **Age & BMI** (continuous and categorical values).
- Questions (3–5) cover **parents’ education, work, and family income**.
- Questions (6–7) cover **interest in health and body weight management**.
- This structure ensures that once the questionnaire is completed, the results were tabulated into the same format as **Table (1)**.

**Tool II: Semi-Structured Interview Guide: *Dining Habits:***

To complement the quantitative questionnaire on fast-food consumption, a short qualitative interview was conducted with a small subset of adult student among university women. The purpose was to explore their dining habits, reasons for food choices, and perceptions of health in more depth:

| Main Question | Objective | Possible Probes / Follow-up Questions |
| --- | --- | --- |
| Can you describe your usual eating habits during a typical week? | To explore general patterns of dining habits. | - How many meals do you usually have per day?  - Do you often skip meals? Why? |
| How often do you eat fast food? | To understand frequency and reasons for fast-food consumption. | - Daily, weekly, or occasionally?  - What influences your choice (taste, time, cost)? |
| At what times of the day do you usually eat fast food? | To identify timing of fast-food consumption. | - Breakfast, lunch, dinner, or snacks?  - Is this linked to your schedule (e.g., classes, exams)? |
| Who do you usually eat with? | To explore social influences on dining habits. | - Do you prefer eating alone or with others?  - How do friends or family influence your food choices? |
| What type of fast food do you usually prefer? | To identify preferences and dietary patterns. | - Sandwiches, fried food, pizza, sweets, etc.?  - Why do you prefer this type? |
| How much do you usually spend on fast food per week? | To examine the economic aspect of fast-food consumption. | - Less than 50 EGP, 50–100 EGP, more than 100 EGP?  - Do you think cost affects your choices? |
| How do you feel after eating fast food? | To explore perceived health impacts and attitudes. | - Do you feel satisfied, guilty, or energized?  - Have you noticed any health effects? |
| What are the main reasons you choose fast food instead of home-cooked meals? | To understand motivational factors. | - Convenience, time-saving, availability, taste? |
| How do you think your fast-food habits affect your health and lifestyle? | To explore awareness of long-term consequences. | - Do you think it affects your weight, energy, concentration? |
| What changes, if any, would you like to make in your dining habits? | To identify readiness for behavioral change. | - Would you prefer to reduce fast food?  - What would help you make these changes? |

✅ Notes: **Clarification Regarding Tool II:**

Regarding tool part 2, a qualitative semi-structured interview guide entitled *Dining Habits* was developed to explore participants’ fast-food consumption practices in depth. The guide included open-ended questions focusing on meal frequency, dining companions, motivations, and perceived barriers. This approach allowed participants to express personal experiences and social contexts beyond structured responses.

**Supplementary Qualitative *Dining Habits* Findings:**
Field notes and semi-structured interviews with a small subset of participants (n = 385) provided narrative comments in response to open-ended questions. A brief thematic review of these responses highlighted additional insights:

- **Patterns of Eating Habits:** Many participants reported having two main meals per day, often skipping breakfast due to busy schedules. A student mentioned: *“I usually skip breakfast because I wake up late before class.”*
- **Frequency and Timing of Fast-Food Consumption:** Fast food was consumed mostly at lunch, especially during university breaks. The majority reported eating fast food two to three times per week, citing taste and time-saving as the main reasons.
- **Social and Economic Influences:** Most participants preferred eating with friends, which often encouraged choosing fast food restaurants near campus. Cost was also mentioned, with average weekly spending ranging between 50–100 EGP.
- **Health Perceptions:** Some participants described feeling satisfied after eating fast food, while others expressed guilt and concern about weight gain.
- **Readiness for Change:** A number of students indicated a desire to reduce fast-food intake, but highlighted time pressure and lack of alternatives as barriers.
